# Circulating miR-133a-3p defines a low-risk subphenotype in patients with heart failure and central sleep apnea: a decision tree machine learning approach

**DOI:** 10.1186/s12967-023-04558-w

**Published:** 2023-10-20

**Authors:** David de Gonzalo-Calvo, Pablo Martinez-Camblor, Thalia Belmonte, Ferran Barbé, Kevin Duarte, Martin R. Cowie, Christiane E. Angermann, Andrea Korte, Isabelle Riedel, Josephine Labus, Wolfgang Koenig, Faiez Zannad, Thomas Thum, Christian Bär

**Affiliations:** 1 Translational Research in Respiratory Medicine, IRBLleida, University Hospital Arnau de Vilanova and Santa Maria, Lleida, Spain; 2 CIBER of Respiratory Diseases (CIBERES), Institute of Health Carlos III, Madrid, Spain; 3 Anesthesiology Department, Geisel School of Medicine at Dartmouth, Hanover, NH USA; 4 Faculty of Health Sciences, Universidad Autonoma de Chile, Providencia, Chile; 5 INSERM 1433, CHRU de Nancy, Centre d’Investigations Cliniques Plurithématique, Institut Lorrain du Cœur et des Vaisseaux, Université de Lorraine, Nancy, France; 6 Department of Cardiology, Royal Brompton Hospital (Guy’s & St Thomas’s NHS Foundation Trust), London, UK; 7 Comprehensive Heart Failure Center, University and University Hospital Würzburg, Würzburg, Germany; 8 Department of Medicine I, University Hospital Würzburg, Würzburg, Germany; 9 Institute of Molecular and Translational Therapeutic Strategies (IMTTS), Hannover Medical School, Carl-Neuberg-Str. 1, 30625 Hannover, Germany; 10 Cellular Neurophysiology, Hannover Medical School, Carl-Neuberg-Str. 1, 30625 Hannover, Germany; 11 Deutsches Herzzentrum München, Technische Universität München, Munich, Germany; 12 German Centre for Cardiovascular Research (DZHK), Partner Site Munich Heart Alliance, Munich, Germany; 13 Institute of Epidemiology and Medical Biometry, University of Ulm, Ulm, Germany; 14 Université de Lorraine, Inserm, Centre d’Investigations Cliniques-Plurithématique 1433, Inserm U1116, CHRU Nancy, F-CRIN INI-CRCT Network, Nancy, France; 15 Fraunhofer Institute for Toxicology and Experimental Medicine (ITEM), Nikolai-Fuchs-Str. 1, 30625 Hannover, Germany

**Keywords:** Biomarker, Central sleep apnea, Decision tree learning, Heart failure, Machine learning, microRNA, Reduced ejection fraction, SERVE-HF

## Abstract

**Background:**

Patients with heart failure with reduced ejection fraction (HFrEF) and central sleep apnea (CSA) are at a very high risk of fatal outcomes.

**Objective:**

To test whether the circulating miRNome provides additional information for risk stratification on top of clinical predictors in patients with HFrEF and CSA.

**Methods:**

The study included patients with HFrEF and CSA from the SERVE-HF trial. A three-step protocol was applied: microRNA (miRNA) screening (n = 20), technical validation (n = 60), and biological validation (n = 587). The primary outcome was either death from any cause, lifesaving cardiovascular intervention, or unplanned hospitalization for worsening of heart failure, whatever occurred first. MiRNA quantification was performed in plasma samples using miRNA sequencing and RT-qPCR.

**Results:**

Circulating miR-133a-3p levels were inversely associated with the primary study outcome. Nonetheless, miR-133a-3p did not improve a previously established clinical prognostic model in terms of discrimination or reclassification. A customized regression tree model constructed using the Classification and Regression Tree (CART) algorithm identified eight patient subphenotypes with specific risk patterns based on clinical and molecular characteristics. MiR-133a-3p entered the regression tree defining the group at the lowest risk; patients with log(NT-proBNP) ≤ 6 pg/mL (miR-133a-3p levels above 1.5 arbitrary units). The overall predictive capacity of suffering the event was highly stable over the follow-up (from 0.735 to 0.767).

**Conclusions:**

The combination of clinical information, circulating miRNAs, and decision tree learning allows the identification of specific risk subphenotypes in patients with HFrEF and CSA.

**Supplementary Information:**

The online version contains supplementary material available at 10.1186/s12967-023-04558-w.

## Introduction

Patients with symptomatic chronic heart failure (CHF) show a high prevalence of sleep-disordered breathing (SDB) [1]. In particular, central sleep apnea (CSA) can be found in up to 40% of patients with HF with reduced ejection fraction (HFrEF) [2]. Patients with HFrEF and CSA represent a population at very high risk of adverse outcomes [3, 4]. Additional efforts focused on risk stratification are imperative in order to facilitate care and optimal treatment allocation. In this context, the retrospective analysis of biosamples from the SERVE-HF (Treatment of Sleep-Disordered Breathing with Predominant Central Sleep Apnea by Adaptive Servo Ventilation in Patients with Heart Failure) trial employing state-of-the-art molecular and computational tools constitute an outstanding platform to develop novel biological markers [5–7].

In the last decade, microRNAs (miRNAs), single-stranded small RNA molecules of 19–25 nucleotides in length that regulate gene expression at a posttranscriptional level, have gained great attention as biomarkers with potential clinical application [8]. The noninvasive quantification of miRNA profiles in different bodily fluids has been reported to be highly sensitive, robust and cost-effective for the clinical management of different pathological conditions, including HF [9]. In this context, the combination of miRNA profiling and clinical data using machine learning (ML) algorithms has recently become an innovative approach to define patient subphenotypes [10]; and consequently, a novel strategy towards precision medicine [11].

The number of studies in the field of miRNAs and SDB is limited [12]. Furthermore, no previous studies have attempted to use miRNAs for risk stratification in the field of CSA. Here, we hypothesized that the circulating miRNome may aid in risk stratification of the patients with HFrEF and CSA. Therefore, the aim of the current study is to investigate the potential of plasma miRNAs as biomarkers of adverse outcomes in this patient population, specifically, whether these transcripts can improve the accuracy of a clinical prognostic model previously described in the same study population [13].

## Methods

### Study design

The SERVE-HF trial investigated the effects of adding adaptive servo ventilation (ASV) (AutoSet CS, ResMed) vs. guideline-based medical treatment alone on survival and cardiovascular outcomes in patients with HFrEF and predominantly CSA. The SERVE-HF trial was an international, multicenter, randomized, parallel-group and event-driven study (clinical trial identifier: NCT00733343). Information about the trial design, procedures, outcomes, and results have been previously reported [5, 6]. The trial was conducted in accordance with the Good Clinical Practice guidelines and principles of the 2002 Declaration of Helsinki. Institutional Review Board approval was obtained, and all patients signed an informed consent form to participate in the study.

### Study patients

Enrolled patients had HF with a left ventricular ejection fraction (LVEF) ≤ 45%, New York Heart Association (NYHA) class ≥ II, and predominant CSA (apnea–hypopnea index [AHI] ≥ 15 events per hour, with > 50% central events and a central AHI of ≥ 10 events per hour). Patients were advised to use the ASV device for at least 5 h per night, 7 days per week.

This sub-study includes 587 patients available for miRNA analysis (289 in the control group and 298 in the ASV group). Plasma samples were collected at the time of randomization and stored at  − 80  C for later analysis.

### Outcome

The primary outcome in the time-to-event analysis was the first event of the composite of death from any cause, a life-saving cardiovascular intervention, or an unplanned hospitalization for worsening chronic heart failure, with the latter two end-point events being assessed by the end-point review committee. Life-saving cardiovascular interventions included heart transplantation, implanting a long-term ventricular assist device, resuscitation after sudden cardiac arrest, or appropriate shock for ventricular arrhythmia in patients with an implantable cardioverter-defibrillator. Previous results from the SERVE-HF trial showed that the incidence of the primary outcome did not differ significantly between the control and ASV groups [6].

### microRNA quantification

MiRNA quantification was performed blinded to clinical variables. The results were merged with the baseline clinical data. In the screening phase, miRNA expression profiles were assessed using the HTG EdgeSeq miRNA Whole Transcriptome Assay (miRNA WTA) (HTG Molecular, Tucson, AZ, USA). This technique is a multiplexed, digital expression assay that combines quantitative nuclease protection (qNPA) with next-generation sequencing (NGS) to quantify miRNA expression without RNA extraction. The HTG assay includes probes for 2083 distinct human miRNAs from miRBase v20 [14] as well as probes for 13 housekeeping mRNAs, 5 negative process controls, and one positive process control. Sequencing was performed on the Illumina MiSeq platform.

For validation, total RNA was isolated from 150 µL of plasma using the miRNeasy Serum/Plasma Kit (Qiagen, Hilden, Germany) according to the manufacturer’s protocol. Synthetic *Caenorhabditis elegans* cel-miR-39-3p (1.6 × 10^8^ copies/µL) (Qiagen) was added to each sample as a quality control for the RNA isolation procedure. RNA was stored at – 80 ºC. Reverse transcription was performed using the Reverse Transcription TaqMan MicroRNA Reverse Transcription Kit (Applied Biosystems^®^, Darmstadt, Germany). The reverse transcription reaction was then diluted with water (1:3 ratio). The expression of miRNAs which met the selection criteria were analyzed by quantitative RT-PCR (RT‐qPCR) with specific TaqMan miRNA assays (Applied Biosystems^®^). Amplification was performed using the ViiA 7 Real-Time PCR System (Applied Biosystems^®^). Relative miRNA expression was calculated by first exporting the raw amplification data to the LinRegPCR (11.0) software which calculates the initial concentration (N0) of a given miRNA per sample [15, 16], expressed in arbitrary fluorescence units. Relative miRNA levels (RQ) were then calculated as: RQ = N0[miRNA]/N0[miR-486-5p], using miR-486-5p as an internal standard [7]. Relative expression levels were log-transformed for statistical analysis.

### Statistical analysis

All analyses were performed with R software version 4.0.3 (the R foundation for Statistical Computing). The two-tailed significance level was set at p < 0.05. Continuous variables were expressed as mean ± standard deviation (SD) and median (interquartile range), and categorical variables were expressed as frequencies (percentages). Comparisons of baseline characteristics and miRNA levels between study groups were carried out using non-parametric Wilcoxon test for continuous variables and Fisher’s exact test for categorical variables. Correlations between continuous variables (baseline characteristics and miRNA variables) were assessed with Spearman rank correlation coefficients.

Unadjusted and adjusted associations of miRNA levels with primary outcome were assessed using Cox regression models. Associated crude and adjusted hazard ratios (HR) with their 95% confidence intervals were presented as HR (95% CI). Three models were constructed using the clinical risk factors identified by Ferreira et al. [13]. Model 1 included ASV, age, and sex. Model 2 included variables from model 1 plus systolic blood pressure (SBP) < 120 mmHg, diabetes, diuretics, cardiac device, and 6-min walking distance, in addition to atrial fibrillation. Model 3 included variables from model 2 and log(NT-proBNP). The goodness of fit of the models was assessed by calculating Harrell’s c-index. The prognostic capacity of each miRNA to predict the event on top of clinical model during follow-up was assessed by calculating the increase in c-index, the continuous Net Reclassification Improvement (cNRI) and the Integrated Discrimination Improvement (IDI). cNRI and IDI were calculated using the survIDINRI package of the R software [17].

The customized regression tree model was constructed using the Classification and Regression Trees (CART) algorithm [18] and implementing HR as splitting criteria (the flowchart for the customized-CART algorithm is shown in Additional file 1: Figure S1). A bagging procedure (based on 1000 iterations) was used for variable selection and error measurement [19]. The goal of this model was to split the population into risk groups aiming to predict the average risk of having the event, rather predicting whether or not the participants will suffer the studied event over a period of time. In this context, standard confusion matrix and similar error measures do not apply. We use incidence rates (IR) of event per 100 patients/year to summarize the absolute risk. HR were used for representing relative risks within each final node. Kaplan–Meier curves were used to illustrate differences between groups in the observed time-to-event. As measures of classification accuracy, we considered: (1) the incremental area under the cumulative/dynamic receiver-operating characteristic curve (iAUC) between 3 months and 5 years of follow-up [20] of the ordinal risk represented by a hierarchization of end nodes; and (2) the incidence rate variation index (IRV) defined by ${IRV=(1/N){\sum }_{i=1}^{f}n}_{i}|{IR}_{i}-IR|$, where IR is the incidence rate of the population, and IR_i_ and n_i,_ the incidence rate and the sample size on the i-th end node, respectively ($1\le i\le f)$, where $f$ stands for the number of terminal nodes. The obtained results were employed in an informed stepwise Cox regression model. R statistical environment (www.r-project.org) was also used for these statistical analyses including the survival [21], rpart [22], and nsROC [23] packages.

## Results

### Patient characteristics

Patient characteristics are shown in Table 1. The mean age was 69.5 ± 9.8 years and 89.8% were male. The mean ± SD follow-up time was of 3.1 ± 1.8 years, similar to the SERVE-HF biomarker sub-study (Additional file 1: Table S1). Patients with the primary outcome during the follow-up were older, predominately male, and showed a poor clinical and biochemical profile. ASV and control groups were well-balanced (Additional file 1: Table S2), except for central apnea index/total AHI [45% (19–67) vs. 53% (24–76), p-value = 0.018] and for the use of antiarrhythmic drugs, which was higher in the ASV group (21.5% vs. 12.8%, p-value = 0.006). The incidence of the primary outcome did not significantly differ between the ASV group and the control group, with event rates of 56.7% and 49.1%, respectively.

**Table 1:** Baseline characteristic levels according to the primary outcome

Variable	Overall (n = 587)			Patients without primary outcome (n = 276)			Patients with primary outcome (n = 311)			p-value^*^
	N	Mean ± SD/n (%)	Median (Q1–Q3)	N	Mean ± SD/n (%)	Median (Q1–Q3)	N	Mean ± SD/n (%)	Median (Q1–Q3)	
Study intervention group	587			276			311			0.069
Control		289 (49.2%)			147 (53.3%)			142 (45.7%)		
ASV		298 (50.8%)			129 (46.7%)			169 (54.3%)		
Age (years)	587	69.5 ± 9.8	71 (63–77)	276	68.1 ± 9.8	70 (62–76)	311	70.8 ± 9.6	72 (65–78)	**0.0005**
Male	587	527 (89.8%)		276	238 (86.2%)		311	289 (92.9%)		**0.009**
Body mass index (kg/m^2^)	582	28.7 ± 5.0	28.1 (25.2–31.1)	272	29.1 ± 5.2	28.4 (25.4–31.7)	310	28.4 ± 4.8	27.8 (25.1–30.7)	0.13
NYHA class III/IV	583	421 (72.2%)		273	180 (65.9%)		310	241 (77.7%)		**0.002**
LVEF (%)	444	33.5 ± 7.7	35 (29–40)	217	35.4 ± 7.2	36 (30–40)	227	31.8 ± 7.6	31 (25–38)	** < 0.0001**
Diabetes	583	243 (41.7%)		272	88 (32.4%)		311	155 (49.8%)		** < 0.0001**
Ischemic HF	572	326 (57.0%)		271	140 (51.7%)		301	186 (61.8%)		**0.018**
Systolic blood pressure (mmHg)	577	124.3 ± 19.9	120 (110–140)	270	127.1 ± 19.3	125 (110–140)	307	121.8 ± 20.1	120 (110–135)	**0.0005**
Left bundle-branch-block	573	150 (26.2%)		268	58 (21.6%)		305	92 (30.2%)		**0.022**
Atrial fibrillation	574	176 (30.7%)		268	69 (25.7%)		306	107 (35.0%)		**0.018**
Cardiac device	587	313 (53.3%)		276	119 (43.1%)		311	194 (62.4%)		** < 0.0001**
Hemoglobin (g/dL)	577	13.9 ± 1.6	14.1 (12.9–15.0)	272	14.2 ± 1.4	14.3 (13.3–15.2)	305	13.7 ± 1.7	13.8 (12.6–14.9)	**0.001**
eGFR CKD-EPI formula (mL/min/1.73m^2^)	570	57.1 ± 21.0	55.8 (40.6–72.8)	266	63.0 ± 19.8	63.1 (48.1–78.1)	304	52.0 ± 20.7	50.7 (35.1–65.3)	** < 0.0001**
6-min walk distance (meters)	558	329.0 ± 125.0	340 (250–423)	262	369.6 ± 109.5	380 (300–450)	296	293.0 ± 127.0	300 (200–382)	** < 0.0001**
ACEI or ARB	587	540 (92.0%)		276	260 (94.2%)		311	280 (90.0%)		0.069
Beta-blocker	587	537 (91.5%)		276	255 (92.4%)		311	282 (90.7%)		0.55
Aldosterone antagonist	587	293 (49.9%)		276	137 (49.6%)		311	156 (50.2%)		0.93
Diuretic	587	509 (86.7%)		276	219 (79.3%)		311	290 (93.2%)		** < 0.0001**
Cardiac glycoside	587	150 (25.6%)		276	55 (19.9%)		311	95 (30.5%)		**0.003**
Antiarrhythmic drug	587	101 (17.2%)		276	35 (12.7%)		311	66 (21.2%)		**0.006**
Epworth Sleep Scale score	586	6.9 ± 4.4	6 (4–9)	275	6.5 ± 4.2	6 (3–9)	311	7.2 ± 4.5	6 (4–10)	0.073
AHI (n events/hr)	586	30.3 ± 12.7	27 (20–38)	276	31.4 ± 13.4	29 (21–39)	310	29.2 ± 12.0	27 (19–38)	0.070
Central apnea index/total AHI (%)	586	47.4 ± 29.4	48 (22–73)	276	45.9 ± 30.2	45 (19–73)	310	48.8 ± 28.6	50 (25–74)	0.24
Central AHI/total AHI (%)	586	80.7 ± 15.0	84 (69–93)	276	80.6 ± 15.5	84 (68—93)	310	80.8 ± 14.5	83 (70–93)	0.95
Oxygen desaturation index	585	33.2 ± 17.4	31 (21–44)	275	32.9 ± 16.7	31 (21–44)	310	33.4 ± 18.1	32 (20–44)	0.87
Average oxygen saturation (%)	587	92.8 ± 2.4	93 (92–94)	276	93.1 ± 2.2	93 (92—95)	311	92.5 ± 2.4	93 (91—94)	**0.0006**
Minimum oxygen saturation (%)	586	80.9 ± 6.6	82 (78–85)	276	81.6 ± 6.5	83 (78–86)	310	80.2 ± 6.7	82 (77–85)	**0.004**
Time with oxygen saturation < 90% (min)	584	48.4 ± 63.6	22 (5–65)	276	43.0 ± 64.0	17 (3–54)	308	53.3 ± 62.9	29 (8–76)	**0.0004**
Cheyne-stokes respiration	506			241			265			0.68
< 20%		106 (20.9%)			51 (21.2%)			55 (20.8%)		
20–50%		191 (37.7%)			95 (39.4%)			96 (36.2%)		
> 50%		209 (41.3%)			95 (39.4%)			114 (43.0%)		
NT-proBNP (pg/mL)	583	2717 ± 4691	1400 (605–3036)	273	1788 ± 3523	881 (354–1828)	310	3536 ± 5392	2044 (975–3757)	** < 0.0001**
Primary outcome	587	311 (53.0%)		276	0 (0.0%)		311	311 (100.0%)		** < 0.0001**
Time to primary outcome (years)	587	2.3 ± 1.8	2.1 (0.7–3.6)	276	3.3 ± 1.8	3.3 (2.1–4.7)	311	1.5 ± 1.3	1.0 (0.4–2.2)	** < 0.0001**
Time to follow-up (years)	587	3.1 ± 1.8	3.0 (1.9–4.4)	276	3.3 ± 1.8	3.3 (2.1–4.7)	311	2.9 ± 1.7	2.7 (1.5–4.2)	**0.003**

Bold values indicate the statistically significant results

*N* number of available values, *SD* standard deviation, *Q1* first quartile, *Q3* third quartile, *ACEI* angiotensin-converting enzyme inhibitors, *AHI* apnea hypopnea index, *ARB* angiotensin II receptor blockers, *ASV* adaptive-servo ventilation, *CV* cardiovascular, *HF* heart failure, *LVEF* left ventricular ejection fraction, *NYHA* *class* New York Heart Association

^*^p-value from Wilcoxon test for continuous variables, Fisher's exact test for categorical variables

### MicroRNA sequencing and technical validation

**Fig. 1:**
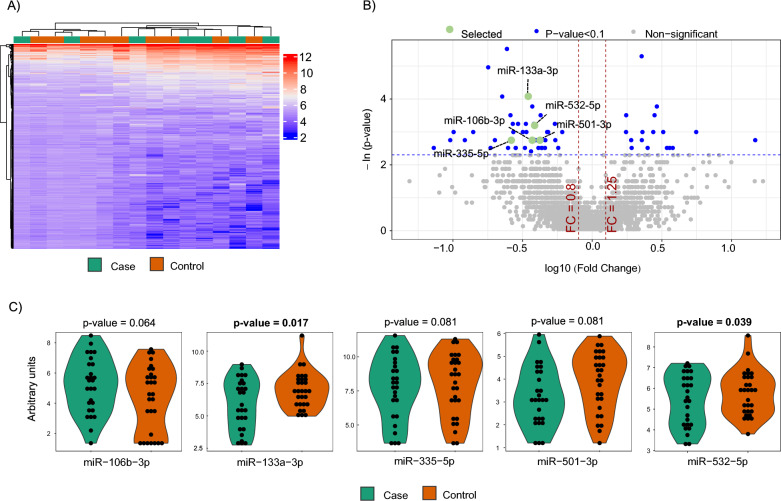
microRNA screening and selection of candidates. **A** Screening phase: Heatmap showing the unsupervised hierarchical clustering in 10 cases and 10 matched controls. The heat map illustrates the levels of plasma microRNAs. Each column represents a patient. Each row represents a microRNA. Red spectra represent increasing expression, while blue spectra represent decreasing expression (see color scale on the right side of the map); **B** Screening phase: Volcano plot for each microRNA after comparison of cases and controls. The log10 (Fold Change) indicates the mean expression level for each microRNA. Each dot represents one microRNA. In green, microRNA candidates that fulfil the selection criteria. In this phase, microRNA expression profiles were assessed using the HTG EdgeSeq miRNA Whole Transcriptome Assay (miRNA WTA) (HTG Molecular, Tucson, AZ, USA); **C** Technical validation phase: Dot plot of microRNA expression validated in 30 cases and 30 matched controls. Comparisons of microRNA levels were performed using non-parametric Wilcoxon test. In this phase, microRNA expression profiles were assessed using RT-qPCR. Relative quantification was performed using miR-486-5p for normalization. Relative expression levels were log-transformed for statistical analysis

MiRNA sequencing was performed in 10 cases and 10 matched controls (Fig. 1A). Patient characteristics are depicted in Additional file 1: Table S3. Four samples did not pass the quality control check and were discarded from subsequent analysis. MiRNA candidates were selected according to the following criteria: ≥ 1.25-fold differential expression, test significance cutoff of p-value less than 0.1, and previously described stable expression in plasma samples [24] in order to ensure their detectability in further analyses. Five miRNAs met these criteria: miR-106b-3p, miR-133a-3p, miR-335-5p, miR-501-3p and miR-532-5p (Fig. 1B). Levels of all miRNAs were downregulated in cases compared to controls.

To corroborate the sequencing results, we evaluated the expression levels of the five miRNAs by RT-qPCR in a sample set of 30 cases and 30 matched controls, including the samples used for miRNA sequencing (Additional file 1: Table S4). In this technical validation, four samples did not pass the quality control check and were discarded for further analysis. We retained miRNAs detected in at least 80% of samples, with a test significance cutoff of p-value less than 0.1, showing the same relationship between cases and controls as in the screening phase. All miRNAs were expressed in 100% of the samples. Three out of five miRNAs (miR-106b-3p, miR-335-5p and miR-532-5p) did not show statistical differences between the study groups (Fig. 1C) and were therefore discarded for further biological validation. In concordance with the sequencing findings, the RT-qPCR analysis showed that plasma levels of miR-133a-3p and miR-501-3p were downregulated in the event group (Fig. 1C).

### Biomarker performance

We then quantified miR-133a-3p and miR-501-3p in the whole population using RT-qPCR. No differences in the levels of both miRNAs were observed between treatment groups (control vs. ASV) (Additional file 1: Table S2). During follow-up, a higher proportion of patients with a reported event below the median was observed for miR-133a-3p (59.2% vs. 46.9%, p-value = 0.004) (Table 2). No differences were observed for miR-501-3p (Additional file 1: Table S5). In the multivariate analyses, miR-133a-3p was inversely associated with the risk of experiencing the primary outcome in model 1 and model 2 (Table 3). However, this association was attenuated after adjusting for NT-proBNP levels [HR (95% CI 0.96 (0.92–1.00), p = 0.061)] (model 3). Similar results were observed when both treatment groups were analyzed separately (Table 3). No association was observed between miR-501-3p and the outcome (Table 3). Furthermore, no meaningful correlations were observed between miRNA levels and clinical variables (rho < 0.3) (Additional file 1: Figure S2).

**Table 2:** Comparison of baseline characteristics according to median value of miR-133a-3p

Variable	miR-133a-3p ≤ median (n = 292)			miR-133a-3p > median (n = 292)			p-value^*^
	N	Mean ± SD/n (%)	Median (Q1–Q3)	N	Mean ± SD/n (%)	Median (Q1–Q3)	
Study intervention group	292			292			0.93
Control		142 (48.6%)			144 (49.3%)		
ASV		150 (51.4%)			148 (50.7%)		
Age (years)	292	70.4 ± 9.3	72 (65–78)	292	68.5 ± 10.2	70 (62–76)	**0.019**
Male	292	265 (90.8%)		292	259 (88.7%)		0.50
Body mass index (kg/m^2^)	288	28.3 ± 4.8	27.8 (24.7–30.6)	291	29.2 ± 5.2	28.4 (25.7–31.7)	0.027
NYHA class III/IV	289	220 (76.1%)		291	199 (68.4%)		0.041
LVEF (%)	208	33.8 ± 7.5	35 (29–40)	234	33.4 ± 7.7	35 (29–40)	0.43
Diabetes	289	115 (39.8%)		291	127 (43.6%)		0.36
Ischemic HF	280	174 (62.1%)		289	150 (51.9%)		**0.014**
Systolic blood pressure (mmHg)	286	124.8 ± 20.5	120 (110–140)	288	123.9 ± 19.3	120 (110–140)	0.84
Left bundle-branch-block	283	81 (28.6%)		287	68 (23.7%)		0.18
Atrial fibrillation	284	96 (33.8%)		287	79 (27.5%)		0.12
Cardiac device	292	161 (55.1%)		292	151 (51.7%)		0.46
Hemoglobin (g/dL)	285	13.9 ± 1.6	14.0 (12.8–15.0)	289	14.0 ± 1.5	14.2 (13.1–15.0)	0.24
eGFR CKD-EPI formula (mL/min/1.73m^2^)	283	55.6 ± 21.5	55.2 (37.8–72.0)	284	58.8 ± 20.5	56.9 (44.3–73.9)	0.12
6-min walk distance (meters)	276	331.3 ± 118.8	345 (258–420)	279	327.3 ± 131.0	332 (245–438)	0.84
ACEI or ARB	292	273 (93.5%)		292	265 (90.8%)		0.28
Beta-blocker	292	267 (91.4%)		292	267 (91.4%)		1.00
Aldosterone antagonist	292	130 (44.5%)		292	161 (55.1%)		**0.013**
Diuretic	292	255 (87.3%)		292	251 (86.0%)		0.72
Cardiac glycoside	292	78 (26.7%)		292	72 (24.7%)		0.64
Antiarrhythmic drug	292	55 (18.8%)		292	45 (15.4%)		0.32
Epworth Sleep Scale score	292	6.6 ± 4.3	6 (4–9)	291	7.2 ± 4.4	6 (4–10)	0.16
AHI (n events/hr)	291	30.2 ± 12.3	27 (20–38)	292	30.4 ± 13.2	27 (20–38)	0.96
Central apnea index/total AHI (%)	291	46.2 ± 30.1	47 (19–73)	292	48.6 ± 28.6	51 (24–73)	0.32
Central AHI/total AHI (%)	291	80.8 ± 15.4	85 (69–94)	292	80.6 ± 14.6	82 (70–93)	0.65
Oxygen Desaturation index	292	34.0 ± 17.6	33 (21–44)	290	32.4 ± 17.3	30 (20–43)	0.21
Average oxygen saturation (%)	292	92.6 ± 2.4	93 (91–94)	292	92.9 ± 2.3	93 (92–94)	0.051
Minimum oxygen saturation (%)	291	80.6 ± 6.5	82 (77–85)	292	81.1 ± 6.8	83 (78–86)	0.14
Time with oxygen saturation < 90% (min)	289	50.8 ± 64.8	28 (7–68)	292	46.0 ± 62.6	20 (4–63)	0.15
Cheyne-stokes respiration	252			251			0.13
< 20%		44 (17.5%)			62 (24.7%)		
20–50%		98 (38.9%)			92 (36.7%)		
> 50%		110 (43.7%)			97 (38.6%)		
NT-proBNP (pg/mL)	289	3131 ± 5172	1644 (785–3235)	291	2315 ± 4151	1119 (499–2683)	**0.0004**
Primary outcome	292	173 (59.2%)		292	137 (46.9%)		**0.004**
Time to primary outcome (years)	292	2.3 ± 1.9	2.0 (0.5–3.5)	292	2.4 ± 1.8	2.2 (0.8–3.7)	0.16
Time to follow-up (years)	292	3.0 ± 1.9	3.0 (1.7–4.5)	292	3.1 ± 1.7	3.0 (2.0–4.3)	0.38

Bold values indicate the statistically significant results

*N* number of available values, *SD* standard deviation, *Q1* first quartile, *Q3* third quartile, *ACEI* angiotensin-converting enzyme inhibitors, *AHI* apnea hypopnea index, *ARB* angiotensin II receptor blockers, *ASV* adaptive-servo ventilation, *CV* cardiovascular; *HF* heart failure, *LVEF* left ventricular ejection fraction, *NYHA* class, New York Heart Association

^*^p-value from Wilcoxon test for continuous variables, Fisher's exact test for categorical variables

**Table 3:** Association between the primary outcome and circulating microRNAs

Subgroup	miRNA	Univariable model		Model 1^a^		Model 2^b^		Model 3^c^	
		HR (95% CI)	p-value	HR (95% CI)	p-value	HR (95% CI)	p-value	HR (95% CI)	p-value
Overall	miR-133a-3p	0.93 (0.89–0.96)	** < 0.0001**	0.93 (0.90–0.97)	**0.0004**	0.94 (0.90–0.97)	**0.001**	0.96 (0.92–1.00)	0.061
	miR-501-3p	0.97 (0.90–1.04)	0.39	0.98 (0.91–1.05)	0.57	0.92 (0.85–0.99)	0.034	0.96 (0.89–1.04)	0.34
Control	miR-133a-3p	0.91 (0.86–0.96)	**0.002**	0.92 (0.86–0.97)	**0.004**	0.92 (0.86–0.98)	**0.008**	0.95 (0.89–1.01)	0.12
	miR-501-3p	0.92 (0.81–1.04)	0.18	0.92 (0.82–1.04)	0.20	0.89 (0.78–1.02)	0.086	0.91 (0.79–1.04)	0.15
ASV	miR-133a-3p	0.94 (0.89–0.99)	**0.013**	0.94 (0.90–0.99)	**0.023**	0.94 (0.90–0.99)	**0.026**	0.96 (0.91–1.01)	0.13
	miR-501-3p	1.00 (0.91–1.10)	0.95	1.01 (0.92–1.11)	0.83	0.93 (0.84–1.03)	0.15	0.98 (0.88–1.09)	0.73

Bold values indicate the statistically significant results

*HR* hazard ratio, *CI* confidence interval

^a^Model 1 included adaptive-servo ventilation (ASV), age and sex

^b^Model 2 included variables of model 1 and systolic blood pressure (SBP) < 120 mmHg, diabetes, diuretic, atrial fibrillation, cardiac device and 6-min walk distance

^c^Model 3 included variables of model 2 and log (NT-proBNP)

The c-index for both miRNAs was low (0.561 for miR-133a-3p and 0.515 for miR-501-3p) (Additional file 1: Table S6). To further explore the potential role of plasma miRNAs as biomarkers, we evaluated the effect of adding the miRNAs to a clinical prognostic model previously described by our group [13], including the prevalence of atrial fibrillation based on the association with CSA [25]. The addition of miR-133a-3p to models 1 and 2 allowed a significant reclassification of the patients (Table 4). However, the addition of miR-133a-3p did not allow the reclassification of the patients when NT-proBNP was part of the clinical model (model 3) (Table 4).

**Table 4:** Prognostic value of circulating microRNAs on top of clinical models

Subgroup	miRNA	C-index (95% CI)		Improvement in c-index		Reclassification indexes at 2 years			
		Clinical model 1	Clinical model 1 + miRNA	Δc-index (95% CI)	p-value	cNRI (95% CI)	p-value	IDI (95% CI)	p-value
Model 1	miR-133a-3p	0.568 (0.534–0.602)	0.597 (0.562–0.631)	0.029 (0.005; 0.052)	**0.016**	0.277 (0.036; 0.436)	**0.014**	0.020 (0.004; 0.044)	**0.002**
	miR-501-3p	0.574 (0.539–0.609)	0.577 (0.542–0.612)	0.003 (− 0.002; 0.007)	0.21	0.085 (− 0.136; 0.246)	0.42	0.001 (− 0.001; 0.010)	0.37
Model 2	miR-133a-3p	0.678 (0.647—0.709)	0.685 (0.654—0.716)	0.007 (− 0.003; 0.016)	0.17	0.230 (0.022; 0.416)	**0.024**	0.015 (0.001; 0.037)	**0.016**
	miR-501-3p	0.682 (0.650–0.714)	0.686 (0.653–0.718)	0.004 (− 0.003; 0.011)	0.29	0.079 (− 0.100; 0.291)	0.23	0.008 (− 0.000; 0.025)	0.086
Model 3	miR-133a-3p	0.731 (0.703–0.759)	0.734 (0.706–0.761)	0.003 (− 0.002; 0.007)	0.25	0.162 (− 0.195; 0.345)	0.25	0.003 (− 0.002; 0.015)	0.33
	miR-501-3p	0.736 (0.708–0.765)	0.737 (0.708–0.766)	0.001 (− 0.002; 0.003)	0.52	0.089 (− 0.265; 0.304)	0.39	0.001 (− 0.001; 0.009)	0.37

Bold values indicate the statistically significant results

*CI* confidence interval, *cNRI* continuous net reclassification improvement, *IDI* integrated discrimination improvement

^a^Model 1 included ASV, age and sex

^b^Model 2 included variables of model 1 and SBP < 120 mmHg, diabetes, diuretic, atrial fibrillation, cardiac device and 6-min walk distance

^c^Model 3 included variables of model 2 and log(NT-proBNP)

**Fig. 2:**
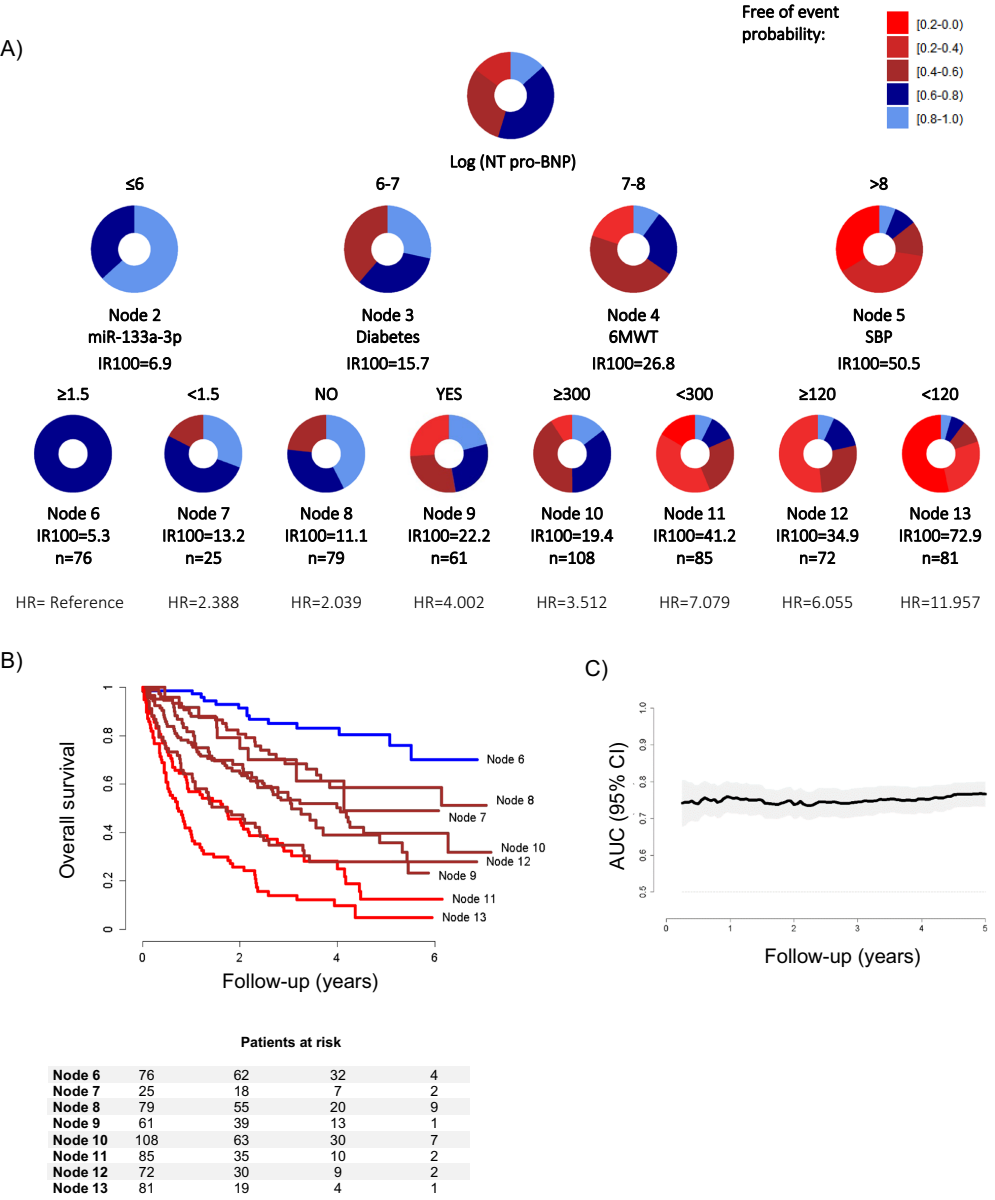
Decision tree machine learning approach. **A** Decision trees calculated using the Classification and Regression Trees (CART) algorithm in the whole study sample. Predictors considered in the analysis were age, treatment group allocation (ASV or control), male sex, SBP < 120 mmHg, diabetes, diuretics, cardiac device and 6 min walk distance, NT‐proBNP, atrial fibrillation, in addition to the microRNA candidate: miR-133a-3p. The results are presented in a binary decision tree that was constructed by splitting a node into two child nodes repeatedly. Generation of novel nodes was based on the selected predictors and cutoffs. Incidence rates (IR) of events per 100 patients/year, number of patients per node and hazard ratios (HR) for the eight final nodes defined by the regression tree model including microRNAs are included. The length of each color in the bands is proportional to the percentage of the total time that patients are submitted to the risk range; **B** Kaplan–Meier curves illustrated differences among nodes in the observed time-to-event outcome. Patients at risk for each subgroup of patients identified are displayed; **C** Incremental area under the cumulative/dynamic ROC curve (iAUC) of the ordinal risk of the final nodes. MicroRNA expression profiles were assessed using RT-qPCR. Relative quantification was performed using miR-486-5p for normalization. Relative expression levels were log-transformed for statistical analyses

Based on previous findings of our group [7], we next evaluated whether the plasma levels of miRNAs could define specific groups of patients at specific risk of adverse outcomes. To this end, we constructed customized regression tree models based on the CART algorithm using HRs derived from Cox regression models as splitting criteria. The variables that composed the model previously constructed in the SERVE-HF cohort [13], age, treatment group allocation (ASV or control), male sex, SBP < 120 mmHg, diabetes, diuretic, cardiac device and 6 min walk distance and NT‐proBNP, in addition to atrial fibrillation, were considered in the stratification process **(**Fig. 2A**)**. The circles represent the entire follow-up, and the different colors represent the probability of the patients in the node to be free-of-events. NT-proBNP was the most relevant predictor. Other clinical variables that entered at the second level were diabetes, 6 min walk distance, and SBP. MiR-133a-3p also entered at the second level of the regression tree, redefining a very low-risk group: those patients with log (NT-proBNP) ≤ 6 pg/mL (cutoff for miR-133a-3p = 1.5 arbitrary units).

Survival Kaplan–Meier curves for the final nodes defined by the regression tree model are shown in Fig. 2B. Four subgroups of patients were identified: low risk (node 6 which included miR-133a-3p; reference), intermediate risk (nodes 7, 8, 9 and 10; HR from 2.039 to 4.002), high risk (nodes 11 and 12; HR from 6.055 to 7.079) and very high risk (node 13; HR = 11.957). The iAUC between 3 months and 5 years was 0.758 (0.724 to 0.788) **(**Fig. 2C**)**. The overall predictive capacity was highly stable over the entire follow-up period (from 0.735 to 0.767). The number of participants with and without an event and the performance metrics at different time points are displayed in Additional file 1: Tables S7, S8.

## Discussion

CSA has been described in up to 40% of CHF patients receiving optimal medication [26]. CSA is associated with impaired cardiac function, poor prognosis, and high risk of death in patients with HF [1]; and has therefore been proposed as a marker of HF severity. Novel prognostic approaches are imperative to improve the clinical management of patients with HFrEF and CSA.

Here, we investigated the circulating miRNome in HFrEF and CSA in order to identify novel tools to improve decision-making. Our first analysis suggests that individual miRNAs are poor biomarkers for risk stratification in a heterogeneous cohort of patients with HFrEF and CSA. Patients with low plasma levels of miR-133a-3p were at a higher risk of adverse outcomes. However, the multivariable analysis showed that the association of this miRNA with the outcome was attenuated after adjusting for NT-proBNP levels. In line with this, miR-133a-3p did not improve the prognostic model based on clinical variables when NT-proBNP was included. Due to the weak correlation between miR-133a-3p and NT-proBNP (rho = − 0.167), it seems that miR-133a-3p does not recapitulate the information provided by NT-proBNP, but that NT-proBNP is simply a better biomarker. These results support previous investigations that suggest at best, only modest improvement in prognostication of novel biomarkers in addition to clinical variables and/or natriuretic peptides [27].

We next hypothesized that miRNAs may be useful to define specific subphenotypes of patients with HFrEF and CSA. To explore this hypothesis, we used decision tree learning based on the CART algorithm, a technique that takes into account high-level interactions of predictors and outcomes and defines subgroups of patients (Additional file 1: Figure S1). These results suggest that miR-133a-3p may serve as a complement to the clinical attributes NT-proBNP, diabetes, 6 min walk distance, and SBP. In particular, miR-133a-3p defines a subphenotype with a very low risk for the clinical outcome that would not have been identified without the inclusion of this circulating miRNA. Importantly, the regression tree did not select relevant clinical variables such as age, sex, or atrial fibrillation, which highlights the prognostic value of miR-133a-3p on top of the information already collected in the electronic health record. Plasma levels of miR-133a-3p appear to be informative when medical history alone does not explain the full complexity of the prognosis.

The final nodes of the decision tree model defined eight subgroups of patients with characteristic clinical and molecular patterns and variable levels of risk. Therefore, the tree may provide a framework to efficiently make clinical decisions, such as adjusting therapeutic decisions and follow-up strategies. For instance, we defined a subgroup of patients with a low-risk status (logNT-proBNP levels ≤ than 6 pg/mL and miR-133a-3p levels ≥ than 1.5 arbitrary units, IR100 = 5.3). Conversely, we identified a subphenotype of very high-risk patients with HFrEF and CSA who may benefit most from intensive monitoring and care (logNT-proBNP levels higher > 8 pg/mL and SBP < 120 mmHg, IR100 = 72.9). In summary, our study suggests that risk assessment in patients with HFrEF and CSA could be performed in a more personalized manner. Further investigations on the benefit of individualizing decision-making according to the patient subphenotypes are essential. These findings highlight the potential of advanced statistical approaches for prognostic enrichment.

ML tailored for biomarker discovery has recently emerged as an alternative to traditional statistical approaches, which are often limited to between-group comparisons and/or linear relationships [28]. Despite their proven value as mechanism-based clinical stratification biomarkers, the exploration of miRNAs in the context of ML is still in its infancy. Nevertheless, we have previously described several combinations of clinical variables and plasma miRNAs that allow the identification of specific clinical subphenotypes in heterogeneous diseases such as coronary artery disease [10], cardiometabolic disease [29], and end-stage renal disease [7], demonstrating the validity of decision tree learning. Interestingly, other subclasses of non-coding RNAs, such as circular RNAs (circRNAs), are not useful in the same context [30], which may indicate the higher value of miRNAs to define specific subgroups of patients over other non-coding transcripts when using decision tree models. Our results are also in line with studies showing that the integration of the cell-free miRNA signature with electronic health data, using ML approaches, constitute an innovative approach to define specific clinical patterns and further develop clinical tools [31].

Although the results are promising, some limitations must be acknowledged. First, this is an exploratory study based on a post hoc analysis in a subpopulation of the SERVE-HF trial. Future verification of the findings should be performed in a prospective multicenter study. The specificity of the SERVE-HF cohort limits this step. Nevertheless, the similarities between our study sample set and the entire SERVE-HF population suggest generalizability of the findings to patients with the characteristics of the clinical trial. Second, owing to the epidemiological factors associated with CSA and with HFrEF, relatively few women were recruited into the study. Third, to date, no standardized method has been established for the detection and quantification of circulating miRNAs in plasma/serum samples, making it difficult to compare expression profiles generated by different quantification strategies. Indeed, the reproducibility of the results obtained by different platforms, e.g., microarrays, RNA sequencing, digital droplet PCR or RT-qPCR, is still a challenge, as demonstrated in the technical validation phase of the current investigation in which we validated two out of five candidates. Fourth, the causal association between plasma miRNAs and the outcome is unclear. Mechanistically, miR-133a-3p is a muscle-specific miRNA that has been implicated in cardiac development, cardiac protection, and regeneration [32], which is supported by our observation that patients with higher cardiovascular risk have lower levels of miR-133a-3p. How higher levels of miR-133a-3p mediate protective responses in patients with low NT-proBNP levels warrants further in vivo and in vitro studies.

## Conclusions

In conclusion, we constructed a simple decision tree model to analyze the risk of adverse outcomes in patients with HFrEF and CSA. This approach emerges as a powerful strategy to improve the clinical assessment in specific subgroups of patients by integrating molecular information, i.e., circulating miRNAs and clinical predictors.

### Supplementary Information

**Additional file 1: Table S1.** Baseline characteristics in patients included and not included in the current investigation. **Table S2.** Baseline characteristics and miRNA levels according to study groups. **Table S3.** Baseline characteristics according to the primary outcome (Screening). **Table S4.** Baseline characteristics according to the primary outcome (technical validation). **Table S5.** Comparison of baseline characteristics according to median value of miR-501-3p. **Table S6.** C-index for miRNAs and NT-proBNP. **Table S7.** Number of participants with event (Positive) within time points (1, 3 and 5 years), and those without event. **Table S8.** Classification metrics at different time points. **Figure S1.** Flow-diagram for the customized-CART algorithm. **Figure S2.** Correlation coefficient maps between microRNA expression levels and baseline clinical characteristics. Correlations between continuous variables were assessed with Spearman rank correlation coefficients, represented as rho values.

## Data Availability

The data that supports the findings of this study are available upon reasonable request from the corresponding author.

## Abbreviations

HFrEF: Heart failure with reduced ejection fraction
CSA: Central sleep apnea
miRNA: MicroRNA
SERVE-HF: Treatment of sleep-disordered breathing with predominant central sleep apnea by adaptive servo ventilation in patients with heart failure
CART: Classification and regression trees
CHF: Chronic heart failure
SDB: Sleep-disordered breathing
ASV: Adaptive servo ventilation
LVEF: Left ventricular ejection fraction
ML: Machine learning
NYHA: New York Heart Association
AHI: Apnea–hypopnea index
qNPA: Quantitative nuclease protection
SD: Standard deviation
cNRI: Continuous net reclassification improvement
SBP: Systolic blood pressure
IDI: Integrated discrimination improvement
iAUC: Incremental area under the cumulative/dynamic receiver-operating characteristic curve
IRV: Incidence rate variation index
