# Supplementary material for: Circulating miR-133a-3p defines a low-risk subphenotype in patients with heart failure and central sleep apnea: a decision tree machine learning approach
Source: J Transl Med. 2023 Oct 20;21:742. doi: 10.1186/s12967-023-04558-w (PMC10588036; doi:10.1186/s12967-023-04558-w)
Supplement: Supplementary file 1 — Additional file 1: Table S1. Baseline characteristics in patients included and not included in the current investigation. Table S2. Baseline characteristics and miRNA levels according to study groups. Table S3. Baseline characteristics according to the primary outcome (Screening). Table S4. Baseline characteristics according to the primary outcome (technical validation). Table S5. Comparison of baseline characteristics according to median value of miR-501-3p. Table S6. C-index for miRNAs and NT-proBNP. Table S7. Number of participants with event (Positive) within time points (1, 3 and 5 years), and those without event. Table S8. Classification metrics at different time points. Figure S1. Flow-diagram for the customized-CART algorithm. Figure S2. Correlation coefficient maps between microRNA expression levels and baseline clinical characteristics. Correlations between continuous variables were assessed with Spearman rank correlation coefficients, represented as rho values. [file 12967_2023_4558_MOESM1_ESM.docx]

**Additional file Information**

# Additional file 1: Table S1: Baseline characteristics in patients included and not included in the current investigation

| **Variable** | **Patients included in the SERVE-HF biomarker substudy (n=809)** | | | | **Patients not included in the current investigation (n=222)** | | | **Patients included in current investigation (n=587)** | | | **p-value^*^** |
| --- | --- | --- | --- | --- | --- | --- | --- | --- | --- | --- | --- |
|  | **N** | **Mean ± SD/n (%)** | **Median (Q1 - Q3)** | **N** | | **Mean ± SD/n (%)** | **Median (Q1 - Q3)** | **N** | **Mean ± SD/n (%)** | **Median (Q1 - Q3)** |  |
| Study intervention group | 809 |  |  | 222 | |  |  | 587 |  |  | 0.94 |
| Control |  | 397 (49.1 %) |  |  | | 108 (48.6 %) |  |  | 289 (49.2 %) |  |  |
| ASV |  | 412 (50.9 %) |  |  | | 114 (51.4 %) |  |  | 298 (50.8 %) |  |  |
| Country | 809 |  |  | 222 | |  |  | 587 |  |  | 0.80 |
| Austria |  | 29 (3.6 %) |  |  | | 7 (3.2 %) |  |  | 22 (3.7 %) |  |  |
| Switzerland |  | 3 (0.4 %) |  |  | | 0 (0.0 %) |  |  | 3 (0.5 %) |  |  |
| Germany |  | 776 (95.9 %) |  |  | | 215 (96.8 %) |  |  | 561 (95.6 %) |  |  |
| Netherlands |  | 1 (0.1 %) |  |  | | 0 (0.0 %) |  |  | 1 (0.2 %) |  |  |
| Age (years) | 809 | 69.3 ± 9.7 | 71 (63 - 76) | 222 | | 68.8 ± 9.6 | 71 (62 - 76) | 587 | 69.5 ± 9.8 | 71 (63 - 77) | 0.25 |
| Male | 809 | 734 (90.7 %) |  | 222 | | 207 (93.2 %) |  | 587 | 527 (89.8 %) |  | 0.14 |
| Body mass index (kg/m²) | 801 | 28.7 ± 4.9 | 28.3 (25.4 - 31.1) | 219 | | 28.8 ± 4.6 | 28.4 (25.5 - 31.2) | 582 | 28.7 ± 5.0 | 28.1 (25.2 - 31.1) | 0.42 |
| NYHA class III/IV | 805 | 589 (73.2 %) |  | 222 | | 168 (75.7 %) |  | 583 | 421 (72.2 %) |  | 0.37 |
| LVEF (%) | 581 | 33.2 ± 7.7 | 35 (29 - 40) | 137 | | 32.3 ± 8.0 | 34 (27 - 38) | 444 | 33.5 ± 7.7 | 35 (29 - 40) | 0.13 |
| Diabetes | 805 | 316 (39.3 %) |  | 222 | | 73 (32.9 %) |  | 583 | 243 (41.7 %) |  | **0.024** |
| Ischemic HF | 791 | 441 (55.8 %) |  | 219 | | 115 (52.5 %) |  | 572 | 326 (57.0 %) |  | 0.26 |
| Systolic blood pressure (mmHg) | 798 | 123.7 ± 19.6 | 120 (110 - 136) | 221 | | 122.2 ± 18.6 | 120 (110 - 130) | 577 | 124.3 ± 19.9 | 120 (110 - 140) | 0.30 |
| Left bundle-branch-block | 794 | 206 (25.9 %) |  | 221 | | 56 (25.3 %) |  | 573 | 150 (26.2 %) |  | 0.86 |
| Atrial fibrillation | 795 | 232 (29.2 %) |  | 221 | | 56 (25.3 %) |  | 574 | 176 (30.7 %) |  | 0.16 |
| Cardiac device | 809 | 437 (54.0 %) |  | 222 | | 124 (55.9 %) |  | 587 | 313 (53.3 %) |  | 0.53 |
| Haemoglobin (g/dL) | 797 | 14.0 ± 1.6 | 14.1 (13.0 - 15.1) | 220 | | 14.2 ± 1.5 | 14.2 (13.1 - 15.3) | 577 | 13.9 ± 1.6 | 14.1 (12.9 - 15.0) | 0.078 |
| eGFR CKD-EPI formula (mL/min/1.73m²) | 790 | 57.9 ± 21.0 | 56.9 (41.9 - 73.6) | 220 | | 60.0 ± 20.8 | 58.7 (45.6 - 74.5) | 570 | 57.1 ± 21.0 | 55.8 (40.6 - 72.8) | 0.097 |
| 6-min walk distance (m) | 771 | 330.9 ± 124.8 | 343 (253 - 420) | 213 | | 336.0 ± 124.6 | 350 (260 - 420) | 558 | 329.0 ± 125.0 | 340 (250 - 423) | 0.53 |
| ACEI or ARB | 809 | 749 (92.6 %) |  | 222 | | 209 (94.1 %) |  | 587 | 540 (92.0 %) |  | 0.37 |
| Beta-blocker | 809 | 748 (92.5 %) |  | 222 | | 211 (95.0 %) |  | 587 | 537 (91.5 %) |  | 0.10 |
| Aldosterone antagonist | 809 | 424 (52.4 %) |  | 222 | | 131 (59.0 %) |  | 587 | 293 (49.9 %) |  | **0.022** |
| Diuretic | 809 | 698 (86.3 %) |  | 222 | | 189 (85.1 %) |  | 587 | 509 (86.7 %) |  | 0.57 |
| Cardiac glycoside | 809 | 201 (24.8 %) |  | 222 | | 51 (23.0 %) |  | 587 | 150 (25.6 %) |  | 0.47 |
| Antiarrhythmic drug | 809 | 143 (17.7 %) |  | 222 | | 42 (18.9 %) |  | 587 | 101 (17.2 %) |  | 0.61 |
| Epworth Sleep Scale score | 804 | 7.0 ± 4.5 | 6 (4 - 9) | 218 | | 7.2 ± 4.7 | 6 (4 - 10) | 586 | 6.9 ± 4.4 | 6 (4 - 9) | 0.51 |
| AHI (n events/hr) | 808 | 30.2 ± 12.5 | 28 (20 - 38) | 222 | | 30.2 ± 12.1 | 28 (20 - 37) | 586 | 30.3 ± 12.7 | 27 (20 - 38) | 0.88 |
| Central apnea index/total AHI (%) | 808 | 48.1 ± 29.3 | 50 (22 - 73) | 222 | | 49.8 ± 29.2 | 51 (24 - 74) | 586 | 47.4 ± 29.4 | 48 (22 - 73) | 0.28 |
| Central AHI/total AHI (%) | 808 | 81.0 ± 15.1 | 84 (70 - 94) | 222 | | 81.8 ± 15.4 | 85 (70 - 95) | 586 | 80.7 ± 15.0 | 84 (69 - 93) | 0.21 |
| Oxygen Desaturation index | 805 | 33.5 ± 18.0 | 32 (20 - 44) | 220 | | 34.2 ± 19.4 | 33 (20 - 46) | 585 | 33.2 ± 17.4 | 31 (21 - 44) | 0.54 |
| Average oxygen saturation (%) | 809 | 92.7 ± 2.4 | 93 (92 - 94) | 222 | | 92.5 ± 2.6 | 93 (91 - 94) | 587 | 92.8 ± 2.4 | 93 (92 - 94) | 0.48 |
| Minimum oxygen saturation (%) | 808 | 80.6 ± 6.8 | 82 (77 - 85) | 222 | | 80.0 ± 7.2 | 81 (76 - 85) | 586 | 80.9 ± 6.6 | 82 (78 - 85) | 0.064 |
| Time with oxygen saturation <90% (min) | 803 | 51.4 ± 66.6 | 24 (5 - 70) | 219 | | 59.4 ± 73.5 | 28 (6 - 90) | 584 | 48.4 ± 63.6 | 22 (5 - 65) | 0.13 |
| Cheyne-Stokes respiration | 702 |  |  | 196 | |  |  | 506 |  |  | 0.25 |
| <20% |  | 145 (20.7 %) |  |  | | 39 (19.9 %) |  |  | 106 (20.9 %) |  |  |
| 20-50% |  | 278 (39.6 %) |  |  | | 87 (44.4 %) |  |  | 191 (37.7 %) |  |  |
| >50% |  | 279 (39.7 %) |  |  | | 70 (35.7 %) |  |  | 209 (41.3 %) |  |  |
| NT-proBNP (pg/mL) | 794 | 2646 ± 4330 | 1396 (605 - 3066) | 211 | | 2451 ± 3127 | 1356 (612 - 3197) | 583 | 2717 ± 4691 | 1400 (605 - 3036) | 0.89 |
| Primary outcome | 809 | 430 (53.2 %) |  | 222 | | 119 (53.6 %) |  | 587 | 311 (53.0 %) |  | 0.94 |
| Time to primary outcome (years) | 809 | 2.4 ± 1.9 | 2.1 (0.6 - 3.7) | 222 | | 2.4 ± 2.0 | 2.1 (0.6 - 4.3) | 587 | 2.3 ± 1.8 | 2.1 (0.7 - 3.6) | 0.78 |
| Time to follow-up (years) | 809 | 3.1 ± 1.8 | 3.0 (1.9 - 4.6) | 222 | | 3.2 ± 2.0 | 3.1 (1.5 - 4.9) | 587 | 3.1 ± 1.8 | 3.0 (1.9 - 4.4) | 0.51 |
| N: number of available values; SD: standard deviation; Q1: first quartile; Q3: third quartile. | | | | | | | | | | | |
| ^*^p-value from Wilcoxon test for continuous variables, Fisher's exact test for categorical variables.  ACEI: angiotensin-converting enzyme inhibitors; AHI: apnea hypoapnea index; ARB: angiotensin II receptor blockers; ASV: Adaptive-Servo Ventilation; CV: cardiovascular; HF: heart failure; LVEF: left ventricular ejection fraction; NYHA class: New York Heart Association | | | | | | | | | | | |

# Additional file: 1: Table S2: Baseline characteristics and miRNA levels according to study groups

| **Variable** | **Overall (n=587)** | | | **Control (n=289)** | | | **ASV (n=298)** | | | **p-value^*^** |
| --- | --- | --- | --- | --- | --- | --- | --- | --- | --- | --- |
|  | **N** | **Mean ± SD/**  **n (%)** | **Median**  **(Q1 - Q3)** | **N** | **Mean ± SD/**  **n (%)** | **Median**  **(Q1 - Q3)** | **N** | **Mean ± SD/**  **n (%)** | **Median**  **(Q1 - Q3)** |  |
| Age (years) | 587 | 69.5 ± 9.8 | 71 (63 - 77) | 289 | 69.5 ± 10.2 | 71 (63 - 77) | 298 | 69.5 ± 9.4 | 71 (64 - 77) | 0.74 |
| Male | 587 | 527 (89.8 %) |  | 289 | 260 (90.0 %) |  | 298 | 267 (89.6 %) |  | 0.89 |
| Body mass index (kg/m²) | 582 | 28.7 ± 5.0 | 28.1 (25.2 - 31.1) | 287 | 28.9 ± 5.5 | 28.0 (25.2 - 31.2) | 295 | 28.5 ± 4.5 | 28.3 (25.2 - 31.0) | 0.83 |
| NYHA class III/IV | 583 | 421 (72.2 %) |  | 287 | 211 (73.5 %) |  | 296 | 210 (70.9 %) |  | 0.52 |
| LVEF (%) | 444 | 33.5 ± 7.7 | 35 (29 - 40) | 219 | 33.7 ± 7.4 | 35 (30 - 40) | 225 | 33.4 ± 7.9 | 35 (29 - 40) | 0.71 |
| Diabetes | 583 | 243 (41.7 %) |  | 287 | 124 (43.2 %) |  | 296 | 119 (40.2 %) |  | 0.50 |
| Ischemic HF | 572 | 326 (57.0 %) |  | 278 | 155 (55.8 %) |  | 294 | 171 (58.2 %) |  | 0.61 |
| Systolic blood pressure (mmHg) | 577 | 124.3 ± 19.9 | 120 (110 - 140) | 283 | 123.9 ± 19.8 | 120 (110 - 138) | 294 | 124.6 ± 20.1 | 121 (110 - 140) | 0.70 |
| Left bundle-branch-block | 573 | 150 (26.2 %) |  | 283 | 74 (26.1 %) |  | 290 | 76 (26.2 %) |  | 1.00 |
| Atrial fibrillation | 574 | 176 (30.7 %) |  | 284 | 77 (27.1 %) |  | 290 | 99 (34.1 %) |  | 0.071 |
| Cardiac device | 587 | 313 (53.3 %) |  | 289 | 152 (52.6 %) |  | 298 | 161 (54.0 %) |  | 0.74 |
| Haemoglobin (g/dL) | 577 | 13.9 ± 1.6 | 14.1 (12.9 - 15.0) | 284 | 14.0 ± 1.6 | 14.1 (13.0 - 15.1) | 293 | 13.9 ± 1.6 | 14.1 (12.9 - 15.0) | 0.63 |
| eGFR CKD-EPI formula (mL/min/1.73m²) | 570 | 57.1 ± 21.0 | 55.8 (40.6 - 72.8) | 280 | 58.0 ± 21.4 | 56.8 (42.7 - 74.2) | 290 | 56.3 ± 20.7 | 55.2 (39.5 - 71.1) | 0.29 |
| 6-min walk distance (m) | 558 | 329.0 ± 125.0 | 340 (250 - 423) | 271 | 331.6 ± 130.7 | 341 (250 - 435) | 287 | 326.5 ± 119.5 | 330 (250 - 419) | 0.32 |
| ACEI or ARB | 587 | 540 (92.0 %) |  | 289 | 269 (93.1 %) |  | 298 | 271 (90.9 %) |  | 0.36 |
| Beta-blocker | 587 | 537 (91.5 %) |  | 289 | 269 (93.1 %) |  | 298 | 268 (89.9 %) |  | 0.19 |
| Aldosterone antagonist | 587 | 293 (49.9 %) |  | 289 | 155 (53.6 %) |  | 298 | 138 (46.3 %) |  | 0.083 |
| Diuretic | 587 | 509 (86.7 %) |  | 289 | 257 (88.9 %) |  | 298 | 252 (84.6 %) |  | 0.14 |
| Cardiac glycoside | 587 | 150 (25.6 %) |  | 289 | 64 (22.1 %) |  | 298 | 86 (28.9 %) |  | 0.072 |
| Antiarrhythmic drug | 587 | 101 (17.2 %) |  | 289 | 37 (12.8 %) |  | 298 | 64 (21.5 %) |  | **0.006** |
| Epworth Sleep Scale score | 586 | 6.9 ± 4.4 | 6 (4 - 9) | 289 | 6.9 ± 4.6 | 6 (4 - 9) | 297 | 6.8 ± 4.2 | 6 (4 - 9) | 0.84 |
| AHI (n events/hr) | 586 | 30.3 ± 12.7 | 27 (20 - 38) | 289 | 31.1 ± 13.6 | 29 (21 - 38) | 297 | 29.5 ± 11.8 | 27 (19 - 38) | 0.25 |
| Central apnea index/total AHI (%) | 586 | 47.4 ± 29.4 | 48 (22 - 73) | 289 | 50.3 ± 29.9 | 53 (24 - 76) | 297 | 44.6 ± 28.6 | 45 (19 - 67) | **0.018** |
| Central AHI/total AHI (%) | 586 | 80.7 ± 15.0 | 84 (69 - 93) | 289 | 81.1 ± 15.2 | 85 (70 - 93) | 297 | 80.3 ± 14.8 | 82 (68 - 93) | 0.48 |
| Oxygen Desaturation index | 585 | 33.2 ± 17.4 | 31 (21 - 44) | 287 | 33.8 ± 18.3 | 32 (20 - 44) | 298 | 32.6 ± 16.6 | 30 (21 - 42) | 0.43 |
| Average oxygen saturation (%) | 587 | 92.8 ± 2.4 | 93 (92 - 94) | 289 | 92.8 ± 2.5 | 93 (91 - 95) | 298 | 92.7 ± 2.2 | 93 (92 - 94) | 0.33 |
| Minimum oxygen saturation (%) | 586 | 80.9 ± 6.6 | 82 (78 - 85) | 289 | 80.5 ± 7.1 | 82 (77 - 86) | 297 | 81.3 ± 6.2 | 82 (78 - 85) | 0.45 |
| Time with oxygen saturation <90% (min) | 584 | 48.4 ± 63.6 | 22 (5 - 65) | 289 | 50.2 ± 65.9 | 20 (4 - 71) | 295 | 46.7 ± 61.3 | 23 (7 - 61) | 0.62 |
| Cheyne-Stokes respiration | 506 |  |  | 252 |  |  | 254 |  |  | 0.17 |
| <20% |  | 106 (20.9 %) |  |  | 55 (21.8 %) |  |  | 51 (20.1 %) |  |  |
| 20-50% |  | 191 (37.7 %) |  |  | 85 (33.7 %) |  |  | 106 (41.7 %) |  |  |
| >50% |  | 209 (41.3 %) |  |  | 112 (44.4 %) |  |  | 97 (38.2 %) |  |  |
| NT-proBNP (pg/mL) | 583 | 2717 ± 4691 | 1400 (605 - 3036) | 287 | 2649 ± 4557 | 1395 (571 - 3092) | 296 | 2783 ± 4823 | 1415 (649 - 2990) | 0.55 |
| miR-133a-3p | 584 | 4.14 ± 3.04 | 4.61 (0.21 - 6.67) | 286 | 4.10 ± 3.00 | 4.65 (0.21 - 6.59) | 298 | 4.17 ± 3.07 | 4.58 (0.21 - 6.75) | 0.71 |
| miR-501-3p | 557 | 12.51 ± 1.55 | 11.54 (11.54 - 13.56) | 269 | 12.42 ± 1.47 | 11.54 (11.54 - 13.14) | 288 | 12.58 ± 1.61 | 11.54 (11.54 - 13.78) | 0.32 |
| Primary outcome | 587 | 311 (53.0 %) |  | 289 | 142 (49.1 %) |  | 298 | 169 (56.7 %) |  | 0.069 |
| Time to primary outcome (years) | 587 | 2.3 ± 1.8 | 2.1 (0.7 - 3.6) | 289 | 2.5 ± 1.9 | 2.2 (0.7 - 3.9) | 298 | 2.2 ± 1.7 | 2.0 (0.7 - 3.3) | 0.054 |
| Time to follow-up (years) | 587 | 3.1 ± 1.8 | 3.0 (1.9 - 4.4) | 289 | 3.2 ± 1.8 | 3.0 (2.0 - 4.5) | 298 | 3.0 ± 1.8 | 2.9 (1.8 - 4.2) | 0.36 |
| N: number of available values; SD: standard deviation; Q1: first quartile; Q3: third quartile. | | | | | | | | | | |
| ^*^p-value from Wilcoxon test for continuous variables, Fisher's exact test for categorical variables.  ACEI: angiotensin-converting enzyme inhibitors; AHI: apnea hypoapnea index; ARB: angiotensin II receptor blockers; ASV: Adaptive-Servo Ventilation; CV: cardiovascular; HF: heart failure; LVEF: left ventricular ejection fraction; NYHA class: New York Heart Association | | | | | | | | | | |

# Additional file 1: Table S3: Baseline characteristics according to the primary outcome (Screening)

| **Variable** | **Patients in the screening phase (n=20)** | | | | **Patients without primary outcome (n=10)** | | | | **Patients with primary outcome (n=10)** | | | **p-value^*^** |
| --- | --- | --- | --- | --- | --- | --- | --- | --- | --- | --- | --- | --- |
|  | **N** | **Mean ± SD/n (%)** | **Median (Q1 - Q3)** | **N** | | **Mean ± SD/n (%)** | **Median (Q1 - Q3)** | **N** | | **Mean ± SD/n (%)** | **Median (Q1 - Q3)** |  |
| Study intervention group | 20 |  |  | 10 | |  |  | 10 | |  |  | 1.00 |
| Control |  | 12 (60.0 %) |  |  | | 6 (60.0 %) |  |  | | 6 (60.0 %) |  |  |
| ASV |  | 8 (40.0 %) |  |  | | 4 (40.0 %) |  |  | | 4 (40.0 %) |  |  |
| Age (years) | 20 | 66.1 ± 10.5 | 69 (58 - 73) | 10 | | 65.4 ± 11.5 | 69 (64 - 73) | 10 | | 66.9 ± 10.1 | 67 (58 - 74) | 0.97 |
| Male | 20 | 18 (90.0 %) |  | 10 | | 9 (90.0 %) |  | 10 | | 9 (90.0 %) |  | 1.00 |
| Diabetes | 20 | 20 (100.0 %) |  | 10 | | 10 (100.0 %) |  | 10 | | 10 (100.0 %) |  | NA |
| Systolic blood pressure (mmHg) | 20 | 119.5 ± 15.2 | 118 (110 - 130) | 10 | | 117.7 ± 8.8 | 118 (115 - 120) | 10 | | 121.4 ± 20.1 | 118 (110 - 130) | 1.00 |
| Atrial fibrillation | 20 | 2 (10.0 %) |  | 10 | | 1 (10.0 %) |  | 10 | | 1 (10.0 %) |  | 1.00 |
| Cardiac device | 20 | 10 (50.0 %) |  | 10 | | 5 (50.0 %) |  | 10 | | 5 (50.0 %) |  | 1.00 |
| 6-min walk distance (m) | 18 | 368.8 ± 108.2 | 398 (306 - 420) | 10 | | 365.5 ± 123.4 | 378 (306 - 420) | 8 | | 373.0 ± 94.0 | 410 (320 - 432) | 0.79 |
| Diuretic | 20 | 16 (80.0 %) |  | 10 | | 8 (80.0 %) |  | 10 | | 8 (80.0 %) |  | 1.00 |
| NT-proBNP (pg/mL) | 20 | 2007 ± 2356 | 1156 (485 - 1989) | 10 | | 1442 ± 1494 | 680 (359 - 2032) | 10 | | 2571 ± 2962 | 1553 (719 - 1946) | 0.25 |
| N: number of available values; SD: standard deviation; Q1: first quartile; Q3: third quartile. | | | | | | | | | | | | |
| ^*^p-value from Wilcoxon test for continuous variables, Fisher's exact test for categorical variables.  ASV: Adaptive-Servo Ventilation | | | | | | | | | | | | |

# Additional file 1: Table S4: Baseline characteristics according to the primary outcome (technical validation)

| **Variable** | **Patients in the technical validation phase (n=60)** | | | | **Patients without primary outcome (n=30)** | | | | **Patients with primary outcome (n=30)** | | | **p-value^*^** | |
| --- | --- | --- | --- | --- | --- | --- | --- | --- | --- | --- | --- | --- | --- |
|  | **N** | **Mean ± SD/n (%)** | **Median (Q1 - Q3)** | **N** | | **Mean ± SD/n (%)** | **Median (Q1 - Q3)** | **N** | | **Mean ± SD/n (%)** | **Median (Q1 - Q3)** |  |  |
| Study intervention group | 60 |  |  | 30 | |  |  | 30 | |  |  | 1.00 | |
| Control |  | 32 (53.3 %) |  |  | | 16 (53.3 %) |  |  | | 16 (53.3 %) |  |  | |
| ASV |  | 28 (46.7 %) |  |  | | 14 (46.7 %) |  |  | | 14 (46.7 %) |  |  | |
| Age (years) | 60 | 68.4 ± 9.8 | 71 (61 - 76) | 30 | | 68.2 ± 11.2 | 71 (64 - 77) | 30 | | 68.6 ± 8.3 | 70 (60 - 75) | 0.76 | |
| Male | 60 | 58 (96.7 %) |  | 30 | | 29 (96.7 %) |  | 30 | | 29 (96.7 %) |  | 1.00 | |
| Diabetes | 60 | 10 (16.7 %) |  | 30 | | 5 (16.7 %) |  | 30 | | 5 (16.7 %) |  | 1.00 | |
| Atrial fibrillation | 60 | 10 (16.7 %) |  | 30 | | 5 (16.7 %) |  | 30 | | 5 (16.7 %) |  | 1.00 | |
| Cardiac device | 60 | 32 (53.3 %) |  | 30 | | 16 (53.3 %) |  | 30 | | 16 (53.3 %) |  | 1.00 | |
| 6-min walk distance (m) | 56 | 324.2 ± 125.1 | 350 (258 - 420) | 29 | | 307.2 ± 128.4 | 320 (240 - 396) | 27 | | 342.6 ± 121.2 | 380 (267 - 429) | 0.19 | |
| Diuretic | 60 | 55 (91.7 %) |  | 30 | | 28 (93.3 %) |  | 30 | | 27 (90.0 %) |  | 1.00 | |
| NT-proBNP (pg/mL) | 60 | 2251 ± 2417 | 1304 (575 - 3448) | 30 | | 1714 ± 1574 | 1041 (482 - 3232) | 30 | | 2789 ± 2968 | 1351 (719 - 3687) | 0.19 | |
| N: number of available values; SD: standard deviation; Q1: first quartile; Q3: third quartile. | | | | | | | | | | | | |  |
| ^*^p-value from Wilcoxon test for continuous variables, Fisher's exact test for categorical variables.  ASV: Adaptive-Servo Ventilation | | | | | | | | | | | | |  |

# Additional file 1: Table S5: Comparison of baseline characteristics according to median value of miR-501-3p

| **Variable** | **miR-501-3p ≤ median (n=380)** | | | **miR-501-3p > median (n=177)** | | | **p-value^*^** |
| --- | --- | --- | --- | --- | --- | --- | --- |
|  | **N** | **Mean ± SD/n (%)** | **Median (Q1 - Q3)** | **N** | **Mean ± SD/n (%)** | **Median (Q1 - Q3)** |  |
| Study intervention group | 380 |  |  | 177 |  |  | 0.58 |
| Control |  | 187 (49.2 %) |  |  | 82 (46.3 %) |  |  |
| ASV |  | 193 (50.8 %) |  |  | 95 (53.7 %) |  |  |
| Age (years) | 380 | 70.0 ± 9.6 | 71 (64 - 77) | 177 | 67.9 ± 10.4 | 70 (62 - 76) | **0.036** |
| Male | 380 | 352 (92.6 %) |  | 177 | 149 (84.2 %) |  | **0.004** |
| Body mass index (kg/m²) | 376 | 28.3 ± 4.6 | 27.9 (25.1 - 30.6) | 176 | 29.5 ± 5.5 | 28.7 (26.0 - 32.0) | 0.020 |
| NYHA class III/IV | 377 | 275 (72.9 %) |  | 176 | 120 (68.2 %) |  | 0.27 |
| LVEF (%) | 296 | 34.2 ± 7.7 | 35 (30 - 40) | 126 | 31.9 ± 7.3 | 32 (27 - 38) | **0.004** |
| Diabetes | 377 | 145 (38.5 %) |  | 176 | 82 (46.6 %) |  | 0.078 |
| Ischemic HF | 368 | 216 (58.7 %) |  | 175 | 88 (50.3 %) |  | 0.079 |
| Systolic blood pressure (mmHg) | 373 | 124.4 ± 20.9 | 120 (110 - 140) | 174 | 124.3 ± 18.0 | 120 (110 - 140) | 0.91 |
| Left bundle-branch-block | 372 | 100 (26.9 %) |  | 171 | 45 (26.3 %) |  | 0.92 |
| Atrial fibrillation | 372 | 108 (29.0 %) |  | 172 | 55 (32.0 %) |  | 0.48 |
| Cardiac device | 380 | 207 (54.5 %) |  | 177 | 94 (53.1 %) |  | 0.78 |
| Haemoglobin (g/dL) | 373 | 13.8 ± 1.6 | 13.9 (12.8 - 15.0) | 174 | 14.2 ± 1.4 | 14.4 (13.4 - 15.1) | **0.002** |
| eGFR CKD-EPI formula (mL/min/1.73m²) | 368 | 56.2 ± 20.8 | 56.0 (39.8 - 71.2) | 172 | 59.7 ± 21.3 | 56.1 (44.5 - 77.6) | 0.13 |
| 6-min walk distance (m) | 359 | 341.9 ± 122.9 | 355 (270 - 430) | 169 | 304.4 ± 127.6 | 312 (200 - 405) | **0.002** |
| ACEI or ARB | 380 | 352 (92.6 %) |  | 177 | 162 (91.5 %) |  | 0.73 |
| Beta-blocker | 380 | 343 (90.3 %) |  | 177 | 164 (92.7 %) |  | 0.43 |
| Aldosterone antagonist | 380 | 196 (51.6 %) |  | 177 | 83 (46.9 %) |  | 0.32 |
| Diuretic | 380 | 325 (85.5 %) |  | 177 | 159 (89.8 %) |  | 0.18 |
| Cardiac glycoside | 380 | 84 (22.1 %) |  | 177 | 51 (28.8 %) |  | 0.090 |
| Antiarrhythmic drug | 380 | 62 (16.3 %) |  | 177 | 37 (20.9 %) |  | 0.19 |
| Epworth Sleep Scale score | 380 | 6.6 ± 4.3 | 6 (4 - 9) | 176 | 7.2 ± 4.4 | 6 (4 - 10) | 0.11 |
| AHI (n events/hr) | 380 | 30.6 ± 12.7 | 28 (20 - 38) | 176 | 29.8 ± 12.9 | 26 (20 - 37) | 0.39 |
| Central apnea index/total AHI (%) | 380 | 46.7 ± 29.5 | 46 (21 - 73) | 176 | 48.9 ± 28.9 | 52 (24 - 74) | 0.39 |
| Central AHI/total AHI (%) | 380 | 80.0 ± 14.8 | 82 (67 - 93) | 176 | 82.5 ± 15.4 | 86 (72 - 96) | **0.029** |
| Oxygen Desaturation index | 378 | 33.9 ± 18.0 | 32 (21 - 44) | 177 | 32.1 ± 16.6 | 30 (20 - 43) | 0.40 |
| Average oxygen saturation (%) | 380 | 92.8 ± 2.3 | 93 (92 - 94) | 177 | 92.7 ± 2.5 | 93 (91 - 94) | 0.71 |
| Minimum oxygen saturation (%) | 379 | 81.0 ± 6.9 | 82 (78 - 86) | 177 | 80.3 ± 6.3 | 82 (77 - 85) | 0.080 |
| Time with oxygen saturation <90% (min) | 377 | 46.4 ± 62.4 | 21 (4 - 62) | 177 | 54.3 ± 68.3 | 26 (7 - 79) | 0.11 |
| Cheyne-Stokes respiration | 333 |  |  | 147 |  |  | 0.39 |
| <20% |  | 72 (21.6 %) |  |  | 30 (20.4 %) |  |  |
| 20-50% |  | 129 (38.7 %) |  |  | 49 (33.3 %) |  |  |
| >50% |  | 132 (39.6 %) |  |  | 68 (46.3 %) |  |  |
| NT-proBNP (pg/mL) | 377 | 2835 ± 4504 | 1439 (607 - 3226) | 176 | 2483 ± 5059 | 1328 (566 - 2446) | 0.13 |
| Primary outcome | 380 | 200 (52.6 %) |  | 177 | 95 (53.7 %) |  | 0.86 |
| Time to primary endpoint (years) | 380 | 2.2 ± 1.8 | 2.1 (0.5 - 3.4) | 177 | 2.6 ± 2.0 | 2.2 (0.9 - 4.0) | 0.055 |
| Time to follow-up (years) | 380 | 2.9 ± 1.7 | 2.8 (1.7 - 4.0) | 177 | 3.6 ± 1.8 | 3.9 (2.1 - 5.0) | **<0.0001** |
| N: number of available values; SD: standard deviation; Q1: first quartile; Q3: third quartile. | | | | | | | |
| ^*^p-value from Wilcoxon test for continuous variables, Fisher's exact test for categorical variables. | | | | | | | |

# Additional file 1: Table S6: C-index for miRNAs and NT-proBNP

| **Subgroup** | **miRNA** | **C-index (95% CI)** | **p-value** |
| --- | --- | --- | --- |
| **Overall** | miR-133a-3p | 0.561 (0.527 - 0.594) | **0.0004** |
|  | miR-501-3p | 0.515 (0.486 - 0.544) | 0.31 |
|  | Log(NT-proBNP) | 0.693 (0.664 - 0.722) | **<0.0001** |
| **Control** | miR-133a-3p | 0.561 (0.510 - 0.613) | **0.020** |
|  | miR-501-3p | 0.529 (0.485 - 0.574) | 0.19 |
|  | Log(NT-proBNP) | 0.713 (0.671 - 0.755) | **<0.0001** |
| **ASV** | miR-133a-3p | 0.565 (0.521 - 0.610) | **0.004** |
|  | miR-501-3p | 0.507 (0.468 - 0.547) | 0.72 |
|  | Log(NT-proBNP) | 0.674 (0.633 - 0.715) | **<0.0001** |
| ASV : Adaptive-Servo Ventilation; CI: confidence interval. | |  |  |

Additional file **1: Table S7. Number of participants with event (Positive) within time points (1, 3 and 5 years), and those without event.**

|  | **1 Year** | | **3 Year** | | **5 Year** | |
| --- | --- | --- | --- | --- | --- | --- |
|  | **Positive** | **Negative** | **Positive** | **Negative** | **Positive** | **Negative** |
| **Node 6** | 1 | 69 | 10 | 45 | 12 | 18 |
| **Node 7** | 2 | 22 | 7 | 10 | 9 | 2 |
| **Node 8** | 9 | 64 | 21 | 34 | 25 | 14 |
| **Node 9** | 11 | 49 | 26 | 24 | 33 | 10 |
| **Node 10** | 24 | 81 | 47 | 43 | 54 | 13 |
| **Node 11** | 35 | 45 | 53 | 17 | 60 | 1 |
| **Node 12** | 24 | 42 | 42 | 16 | 45 | 4 |
| **Node 13** | 46 | 29 | 63 | 8 | 66 | 1 |
| **Total** | 155 | 401 | 269 | 197 | 304 | 63 |

Those without event and without enough follow-up (censored with a follow-time below the considered point) are removed from both the Positive and the Negative groups.

Additional file 1: **Table S8. Classification metrics at different time points.**

|  | **1 Year** | **3 Year** | **5 Year** |
| --- | --- | --- | --- |
| **Positivity criteria** | **Nodes 10, 11, 12,13** | **Nodes 9, 10, 11, 12, 13** | **Nodes 8, 9, 10, 11, 12, 13** |
| **Sensitivity (Recall)** | 83.2% | 85.9% | 93.1% |
| **Specificity** | 50.9% | 45.2% | 31.7% |
| **PPV (Precision)** | 39.6% | 68.1% | 72.7% |
| **PNV** | 89.9% | 70.1% | 48.8% |
| **F1-Score** | 54.0% | 76.0% | 81.7% |
| **Accuracy** | 59.9% | 63.9% | 82.6% |

PPV: Predictive Positive Value. PNV: Predictive Negative Value

Positive criteria define the Nodes predicting patients as positive.

Numbers based on those reported in the previous table.


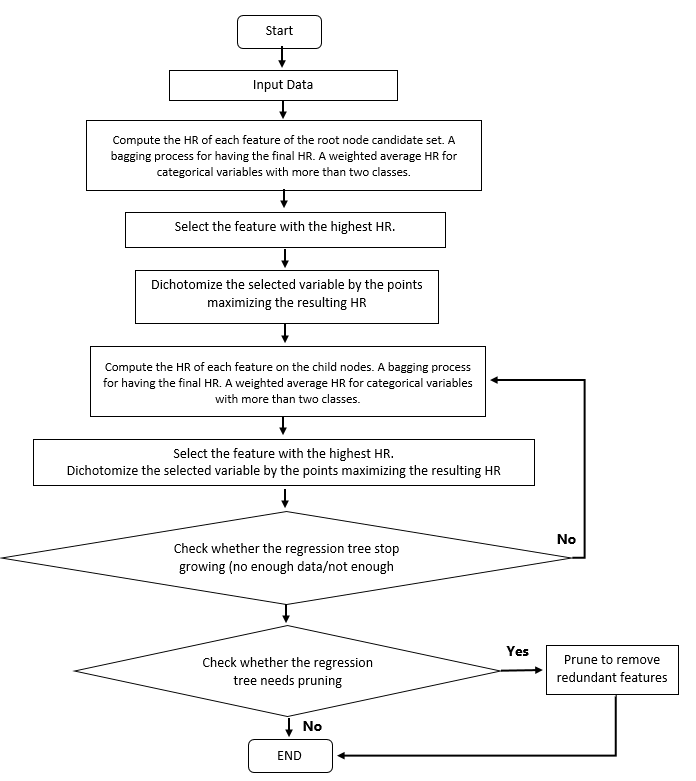


Additional file 1: **Figure S1. Flow-diagram for the customized-CART algorithm.**


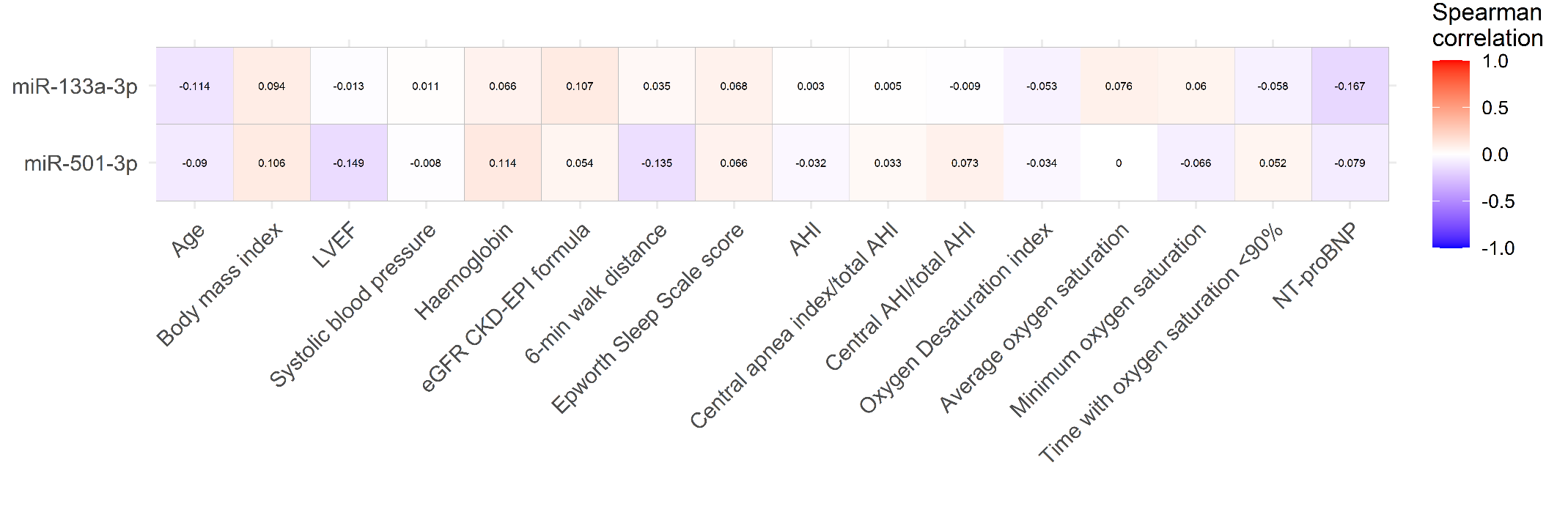


Additional file 1: **Figure S2. Correlation coefficient maps between microRNA expression levels and baseline clinical characteristics.** Correlations between continuous variables were assessed with Spearman rank correlation coefficients, represented as rho values.
